# Use of a Conformational Switching Aptamer for Rapid and Specific Ex Vivo Identification of Central Nervous System Lymphoma in a Xenograft Model

**DOI:** 10.1371/journal.pone.0123607

**Published:** 2015-04-15

**Authors:** Joseph F. Georges, Xiaowei Liu, Jennifer Eschbacher, Joshua Nichols, Michael A. Mooney, Anna Joy, Robert F. Spetzler, Burt G. Feuerstein, Mark C. Preul, Trent Anderson, Hao Yan, Peter Nakaji

**Affiliations:** 1 Division of Neuroscience, Barrow Neurological Institute, St. Joseph’s Hospital and Medical Center, Phoenix, Arizona; 2 Division of Neurological Surgery, Barrow Neurological Institute, St. Joseph’s Hospital and Medical Center, Phoenix, Arizona; 3 Division of Neuropathology, Barrow Neurological Institute, St. Joseph’s Hospital and Medical Center, Phoenix, Arizona; 4 The Biodesign Institute, Arizona State University, Tempe, Arizona; 5 College of Medicine, University of Arizona, Phoenix, Arizona; The Nathan Kline Institute, UNITED STATES

## Abstract

Improved tools for providing specific intraoperative diagnoses could improve patient care. In neurosurgery, intraoperatively differentiating non-operative lesions such as CNS B-cell lymphoma from operative lesions can be challenging, often necessitating immunohistochemical (IHC) procedures which require up to 24-48 hours. Here, we evaluate the feasibility of generating rapid *ex vivo* specific labeling using a novel lymphoma-specific fluorescent switchable aptamer. Our B-cell lymphoma-specific switchable aptamer produced only low-level fluorescence in its unbound conformation and generated an 8-fold increase in fluorescence once bound to its target on CD20-positive lymphoma cells. The aptamer demonstrated strong binding to B-cell lymphoma cells within 15 minutes of incubation as observed by flow cytometry. We applied the switchable aptamer to *ex vivo* xenograft tissue harboring B-cell lymphoma and astrocytoma, and within one hour specific visual identification of lymphoma was routinely possible. In this proof-of-concept study in human cell culture and orthotopic xenografts, we conclude that a fluorescent switchable aptamer can provide rapid and specific labeling of B-cell lymphoma, and that developing aptamer-based labeling approaches could simplify tissue staining and drastically reduce time to histopathological diagnoses compared with IHC-based methods. We propose that switchable aptamers could enhance expeditious, accurate intraoperative decision-making.

## Introduction

Surgical resection of brain tumors is guided by intraoperative frozen section analyses. This technique is useful for diagnosing neoplastic tissue and identifying tumor margins [1]; however, some pathologies that require opposing treatment strategies can be a challenge to diagnose by frozen section alone, and instead require more specific staining. For example, differentiating some gliomas, such as oligodendroglioma or subtypes of high-grade astrocytoma from lymphoma can be challenging with frozen section analysis [2]. Specific diagnostic staining is typically achieved with immunohistochemistry (IHC), a staining process which is too slow to be used intraoperatively [3]. When a diagnosis requires IHC, a biopsy will typically be performed, and definitive treatment for the patient can be delayed by several days while awaiting fixation of tissue, performance of IHC studies, and evaluation by a pathologist for a final pathological diagnosis. Definitive treatment in this situation can vary from a return to the operating room for surgical resection of the lesion in the case of glioma, versus chemoradiation therapy in the case of lymphoma.

IHC was developed during the 1940s by Albert Coons [4]. This histopathological technique generates contrast in cells and tissue by specifically targeting proteins with antibodies. IHC undoubtedly revolutionized the study of tissues in laboratory and clinical settings. With a series of tissue preparation and staining procedures, IHC can provide specific tissue information within 24–48 hours [5, 6]. However, with the advent of new imaging technologies and fluorescent molecular probes, it may be possible to develop protocols for generating IHC-like information in a fraction of the time required by antibodies [7]. If these diagnoses could be made within intraoperative timeframes, they could offer immediate information to the surgeon, who can then make surgical decisions intraoperatively. We sought to design a probe with sufficient speed and accuracy to provide IHC-quality diagnoses in a surgically relevant time frame and to test that probe in brain tumor model systems.

Aptamers are a newer class of target-recognizing molecular probes. They are deoxyribonucleic acid (DNA), ribonucleic acid (RNA), or peptide strands with unique secondary or tertiary structures that can bind to target molecules with high affinity and specificity [8–10]. Aptamers can be generated from a synthetic DNA or RNA pool through an *in vitro* selection method known as systemic evolution of ligands by exponential enrichment [9, 10]. The selected aptamer probes can bind molecular targets with IHC-like specificity in a fraction of the time required by antibodies. Reports have been published which describe aptamers used as recognition molecules for tumor cell identification with various molecular targets [11–13]. In this study, we build from these initial findings to investigate the utility of aptamers for intraoperative histopathological assessments.

Histopathological applications, whether antibody- or aptamer-based, require optimized staining protocols to minimize nonspecific staining. Molecules such as blocking serum, bovine serum albumin, and/or transfer RNA (tRNA) are routinely added during the staining process to reduce nonspecific interactions [14, 15]. Then repetitive washing steps are used to minimize nonspecific staining with aptamers and antibodies. To circumvent this, we developed fluorescently coupled aptamers that are activated upon specific binding-induced conformational change and visualized through the principle of Förster Resonance Energy Transfer (FRET), which has been previously described [16–18]. This can effectively produce an agent that is optically silent until bound and “switches on” after binding to a specific target [16]. This decreases the noise due to nonspecific binding and eliminates the need for multiple time-consuming tissue preparation, staining, and rinsing procedures.

To test whether aptamers specifically label neoplastic brain tissue in a manner that would be practical and timely for clinical intraoperative neuropathological use, we constructed a switchable aptamer complex against a human central nervous system (CNS) lymphoma cell line. We extended a previously reported aptamer [14, 19] to include a fluorophore and a fluorescence quencher to minimize signal in its unbound confirmation. Tested on human tumor cells and fresh tissue from animal models of CNS lymphoma and glioma, we determined whether this probe could rapidly distinguish human lymphoma cells similar to the clinical standard of CD20 immunohistochemistry.

## Materials and Methods

### Preparation of Q-TD05

All DNA oligonucleotides were purchased from Integrated DNA Technologies, CA with high performance liquid chromatography (HPLC) purification. A truncated version of TD05 aptamer, TD05 [17, 19] was used in our study.

The name and sequence of fluorophore-labeled aptamers are as follows:

TD05: 5’ /5Alex488N/AGG AGG ATA GTT AGG TGG CTG TTG AGG GTC TCC TCC TA 3’

Q-TD05: 5’ /5Alex488N/AGG AGG AGA TTT TTT TTT TAG GAG GAT AGT TAG GTG GCT GTT GAG GGT CTC CTC CTA /3BHQ_1/ 3’

Random aptamer (LD20t): 5' FITC-TAG CCA AGG TAA CCA GTA CAA GGT GCT AAA CGT AAT GGC TTC GGC TTA C 3'

### Cell culture

Human glioma cells (U251) and human CNS lymphoma (Ramos) cell lines were acquired from American Type Culture Collection. U251 cells were cultured with Dulbecco’s Modified Eagle Medium media supplemented with 10% fetal bovine serum (FBS), and Ramos cells were cultured in Roswell Park Memorial Institute Medium (RPMI) 1640 supplemented with 10% FBS and 50μM 2-mercaptoethanol (all from Invitrogen, Grand Island, NY). Cells were grown at 37°C in a humidified incubator under 5% CO_2_. In some experiments, tumor cells were infected with a lentivirus to introduce expression of red fluorescent protein (RFP) following the manufacturers’ protocol.

### *In vitro* labeling

The aptamer probes were diluted to 1μM in aptamer binding buffer (6.05 mM Mg^2+^, 1.2 mM Ca 2+, 4.5g/L glucose and 0.2% NaN_3_ in PBS buffer) and heated to 94°C for 5 minutes, followed by immediate chilling on ice for 10 minutes. The annealed aptamers were mixed with yeast tRNA (0.1 mg/mL) to block nonspecific binding and the prepared acute tissue slices were soaked in the above aptamer solution and incubated on ice for 1 hour before confocal imaging.

### Flow cytometry

Cultured U251 cells and Ramos cells were collected and washed twice by the aptamer binding buffer. The annealed aptamers were diluted to 200nM in the binding buffer and added to 5 x 10^5^ cells. The aptamer-cell mixture was incubated on ice for 15 minutes, and analyzed by FACSCalibur from BD Biosciences with or without washing.

### Animals

Crl:NIH-Foxn1^rnu^ rats (5 weeks age) were obtained from The Charles River Laboratories International, Inc. (Wilmington, MA). All experiments were performed in accordance with the guidelines and regulations set forth by the National Institutes of Health Guide for the Care and Use of Laboratory Animals. Experiments were approved by the Institutional Animal Care and Use Committee of the Barrow Neurological Institute at St. Joseph’s Hospital and Medical Center, Phoenix, Arizona.

### Intracranial implantation

Rats were anesthetized by intramuscular injection of 10 mg/kg xylazine and 80 mg/kg ketamine and placed in a small animal stereotactic headframe (Model 900, David Kopf Instruments, Tujunga, CA). A 7-mm incision was made starting between the animal’s eyes to expose bregma. A bur hole was made 3.5 mm lateral to bregma. U251 or Ramos cells were infused at a depth of 4.5 mm below the surface of the brain after the syringe (Hamilton) was advanced 5.0 mm to create a 0.5-mm pocket. The cell suspension was infused using a UMP3-1 UltraMicroPump microinjector (WPI, Sarasota, FL) set to a volume of 10 μL with an infusion rate of 3.00 μL/minute. The needle was withdrawn 2 minutes after the injection to minimize backflow of the cell suspension. The bur hole was covered with bone wax, the skin incision was sutured, and the rats were allowed to recover.

### Acute slices

Twenty-eight days after implantation, rats were deeply anesthetized using the xylazine/ketamine mixture described previously. They were immediately decapitated, and their brains were removed. Coronal slices (350 μm thick) were immediately cut from the cerebral cortex on a Leica VT1200 vibratome in artificial cerebrospinal fluid containing the following (in mM): 126 NaCl, 26 NaHCO_3_, 2.5 KCl, 1.25 NaH_2_PO_4_, 2 MgSO_4_, 2 CaCl_2_ and 10 glucose, pH 7.4. Acute slices were incubated in aptamer solution for 60 minutes.

### Aptamer and CD20 antibody colabeling

Fixed xenograft slices (3 slices from 2 animals) were washed and incubated in anti-CD20 primary antibody (Millipore; 1:250). Sections were then rinsed, incubated with AlexaFluor594 secondary antibody (Invitrogen), rinsed again, and counterstained with Q-TD05 aptamer. Labeled sections were mounted on slides with vectashield (Vector labs) and No 1.5 coverslips (VWR). Total antibody staining time was 24 hours and aptamer staining time was 1 hour.

### Imaging

Aptamer labeled samples were placed in uncoated No.1.5 glass-bottom dishes and positioned on the stage of a Zeiss 710 laser scanning confocal microscope equipped with a 40x/1.2NA water emersion objective. We imaged Q-TD05 by exciting the fluorophore with a 489-nm diode laser and collecting 505nm-535nm emission. We imaged red fluorescent protein by exciting with a 561nm laser and collecting 595nm-625nm emission. Spectral imaging was performed utilizing a 34 channel spectral detector, and separating emission every 10nm between 405nm-745nm. The confocal aperture was set to one Airy unit for imaging. The laser and gain values were set to fill the dynamic range of the photomultiplier tube, and the frame size was set to sample at Nyquist. All post-image processing was conducted with NIH imageJ utilizing linear functions.

## Results

### Development of a FRET-based aptamer for detecting CD20 positive B cells

**Fig 1:**
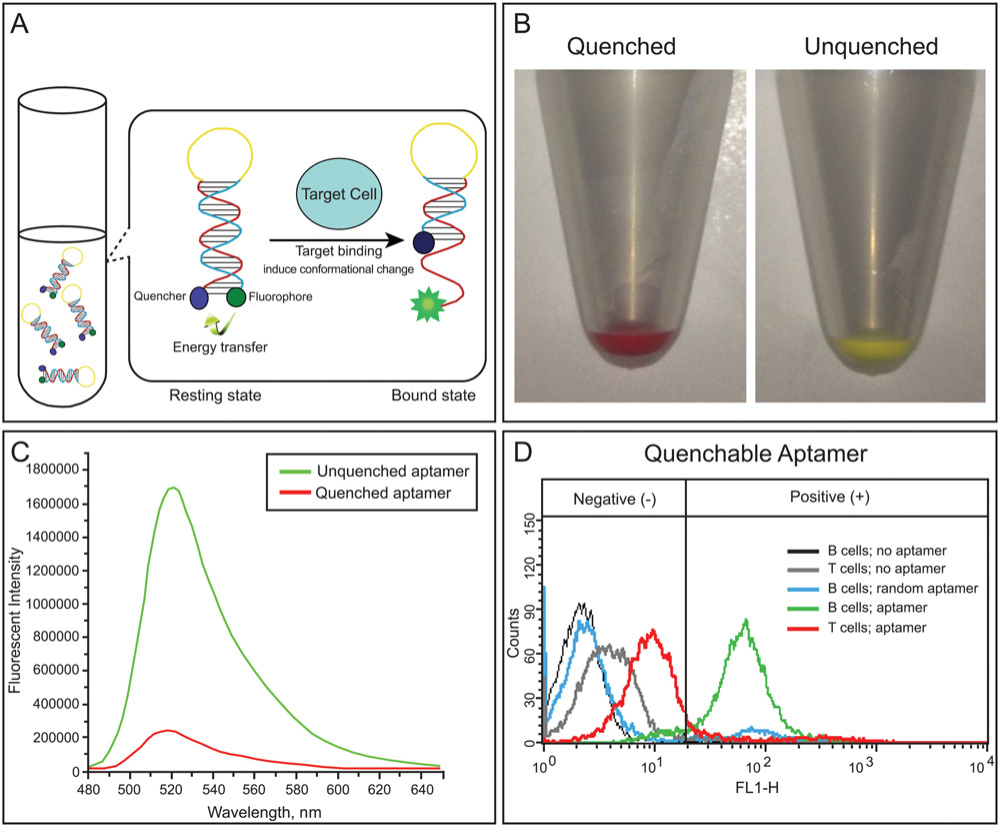
Aptamer fluorescence unquenching. (A, B). A) Illustration of aptamer fluorescence unquenching with binding to molecular target. B) Macrofluorescence change in fluorescence emission between quenched and unquenched aptamers. Target-binding induced change in fluorescence (C, D). C) Fluorescence emission intensity of the unquenched probe versus the quenched. Note a near 8-fold change in fluorescence intensity. D) Fluorescence intensity of the quenchable aptamer tested on negative control T cells and positive control B cells. Note increased fluorescence and number of labeled B cells versus T cells.

Aptamer TD05, which binds to the heavy mu chain of membrane bound immunoglobulin in CD20-positive Burkitt’s lymphoma cells, has been reported previously [14, 19]. In this study, a single stranded DNA linker is added to the existing TD05 aptamer sequence to create the switchable probe “Q-TD05.” The fluorophore, AlexaFluor488, is chemically modified at the 5’ end of this probe, while a Black Hole Quencher (Integrated DNA Technology. Coralville, Iowa) is modified at the 3’ end during chemical synthesis. Conceptually, the aptamer would be minimally fluorescent in its unbound confirmation (designated as “resting” status) and increase in fluorescence after binding to its target (designated as “bound” status; Fig 1A). Under macrofluorescence, we observed the quenched and unquenched aptamer exhibited markedly altered fluorescence (Fig 1B).

The constructed probe was first analyzed by fluorometry to confirm the quenching of the fluorophore (Fig 1C). Here, we included a positive control where the fluorophore was directly labeled at the 5’ end of TD05 aptamer (designated as the “on” TD05 probe). As shown in Fig 1C, the switchable probe in the absence of its target molecule shows a nearly 8 fold decrease in fluorescence intensity compared to the “on” probe in the unquenched confirmation. This indicated the switchable probe is truly in resting status, and is expected to exhibit very low background signal when is used for cell or tissue staining.

To confirm this expectation, we then incubated the aptamer probes on cultured human lymphoma cell lines for 15 minutes and analyzed by flow cytometry (S1 Table). Fluorescence signal caused by nonspecific binding is commonly seen in regular aptamer staining without a washing step. This results in artificial positive cell labeling, especially when applied to tissues. Here, the background staining is compared in the cell lines without washing. The percentage of cells that were artificially labeled in the negative control cell line was used as a parameter to compare the background level (also known as noise) between the “on” probe and the switchable probe. In the group stained with the switchable aptamer probe, the background nonspecific labeling of negative control cells was reduced to about 1/7 (11.77% vs. 81.90% in (Fig 1D) of that in the group stained with the “on” aptamer probe. As an additional control, we incubated B cells with a random fluorescent aptamer and found their fluorescence signal to resemble baseline noise (Fig 1D). This demonstrates that the switchable aptamer could reduce the background staining of non-target cells and could potentially provide specific contrast during tissue staining.

### Quenched TD05 specifically differentiates B cell lymphoma from astrocytoma cells

**Fig 2:**
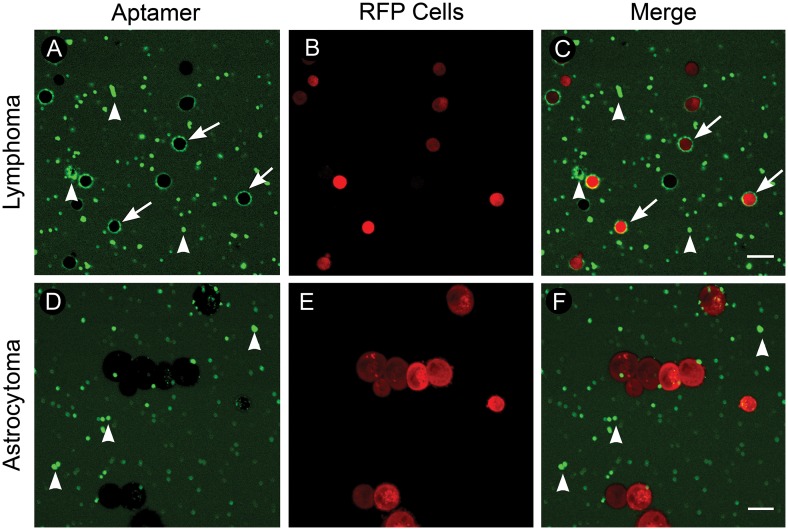
Quenchable aptamer staining of cultured human fluorescent lymphoma and astrocytoma cells. (A-C) Lymphoma. A) B cell lymphoma cells incubated with the quenchable aptamer; note ring-like staining pattern (arrows) and fluorescent artifacts (arrowheads). B) Lymphoma cells expressing RFP. C) Merged image of RFP-lymphoma cells and aptamer staining. (D-F) Astrocytoma. D) Astrocytoma cells incubated with the quenchable aptamer; note fluorescent artifacts (arrowheads) and lack of ring-like staining. E) Astrocytoma cells expressing RFP. F) Merged image of RFP-astrocytoma cells and aptamer staining. Scale bars equal 20*um*. © 2015, Barrow Neurological Institute, provided under CC BY 4.0.

To test whether our aptamer could differentiate clinically meaningful tumor cell types, we tested Q-TD05 on human B cell lymphoma and human astrocytoma cells. Human lymphoma and astrocytoma were first virally transduced with lentivirus to constitutively express red fluorescent protein for specific visualization of tumor cells (Fig 2B and 2E). Fluorescence imaging of lymphoma cells incubated with Q-TD05 revealed a ring-like staining pattern that is typically seen when using CD20 antibody, a common lymphoma marker on lymphoma cells (Fig 2A) [20]. Fluorescence imaging of astrocytoma cells revealed no cell-specific staining (Fig 2D). We noted that Q-TD05 produced some fluorescent artifacts in lymphoma and astrocytoma tissues which differed in morphology from the positive ring-like staining characteristic of CD20 immunostaining (Fig 2 arrowheads). This shows Q-TD05 can differentiate human B cell lymphoma and astrocytoma in cell culture based on fluorescence and staining pattern.

### Comparison of Q-TD05 staining in xenograft acute slices and fixed slices (Quenched TD05 differentiates human B cell lymphoma from astrocytoma in xenograft acute slices)

**Fig 3:**
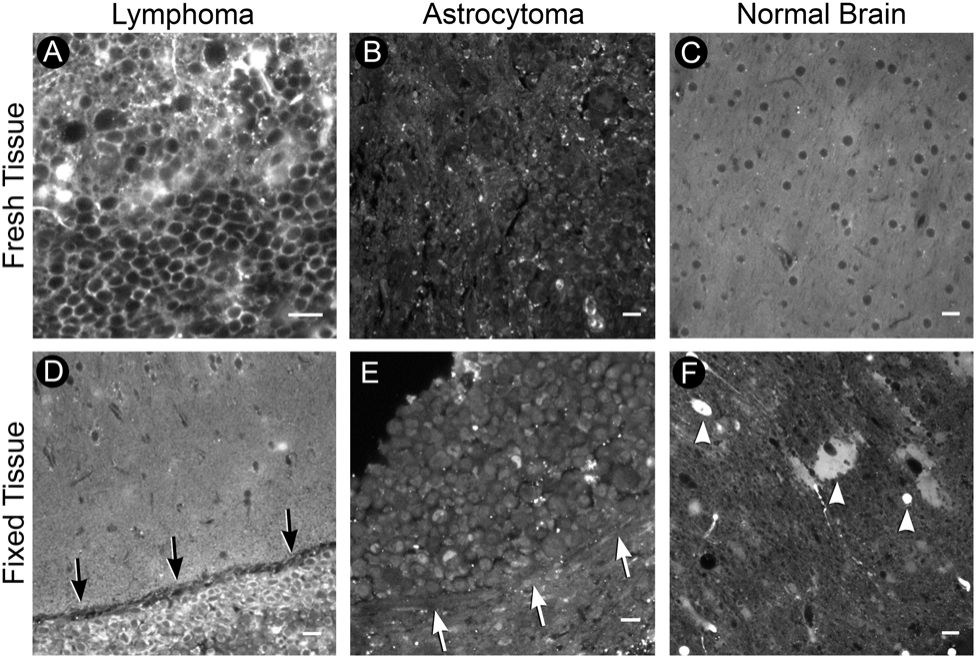
Xenograft acute slices. (A-C) Fresh tissue. A) Tumor region of B cell lymphoma acute slice incubated with the quenchable aptamer. Note the ring-like staining pattern. B) Tumor region of astrocytoma acute slice; note lack of ring-like staining pattern. C) Contralateral normal brain from lymphoma acute slice. Note hypo-fluorescent regions indicating location of cell-bodies. (D-F) Fixed tissue. D) Fixed acute slice containing B cell lymphoma; note positive cells in hypercellular tumor region (arrows). E) Fixed astrocytoma tissue lacking positive staining; note absence of positive cells within hypercellular tumor (arrows). F) Contralateral normal brain from lymphoma fixed slice; note additional fluorescent artifacts (arrowheads). Scale bars equal 20*um*. © 2015, Barrow Neurological Institute, provided under CC BY 4.0.

In order to test Q-TD05 on tissues that more resemble a clinical biopsy, we generated brain slices from rodents intracranially implanted with human B cell lymphoma and astrocytoma cells (n = 12 lymphoma slices from 5 animals and n = 5 astrocytoma slices from 3 animals), and compared the staining patterns. Fresh acute tissue slices containing tumor were incubated with Q-TD05 for one hour and imaged on a confocal microscope. In acute slices containing B cell lymphoma, we found cells with a fluorescent ring-like staining pattern that resembled classic CD20 antibody staining (Fig 3A). On fresh tissue containing human astrocytoma cells we found minimal nonspecific fluorescence and an absence of a ring-like staining pattern (Fig 3B). Much like astrocytoma samples, fresh tissue from normal rodent brain incubated with Q-TD05 only produced weak fluorescence and fluorescence hypointensity of cell bodies (Fig 3C). Next, we examined if active processes were important to the aptamer signaling by examining for similar staining in fixed tissue. We tested Q-TD05 on fixed xenograft brain slices from animals implanted separately with either a B cell lymphoma or astrocytoma. We found a strong fluorescence ring-like staining pattern of cells within lymphoma tumor regions. This was contrasted to weak fluorescence of tissue outside the lymphoma tumor margin (Fig 3D). Fixed astrocytoma slices incubated with Q-TD05 produced only weak fluorescence and were absent of the observed CD20-like staining pattern from fixed lymphoma tissue (Fig 3E). Spectral imaging verified fluorescence signals we detected were fluorophore-specific (S1 Fig). Unique to fixed tissue, we found increased fluorescent artifacts compared to fresh tissue. However, these artifacts did not recapitulate a CD20 circumferential staining pattern (Fig 3F). These results indicate that specific tissue preparation such as fixation is not needed, and may actually reduce the quality, for Q-TD05 staining. Removing time-consuming washing or fixation procedures significantly reduces the complexity of staining and highlights the benefit of the Q-TD05 staining method.

### Quenched TD05 localizes to fluorescent B cell lymphoma cells in xenograft slices

**Fig 4:**
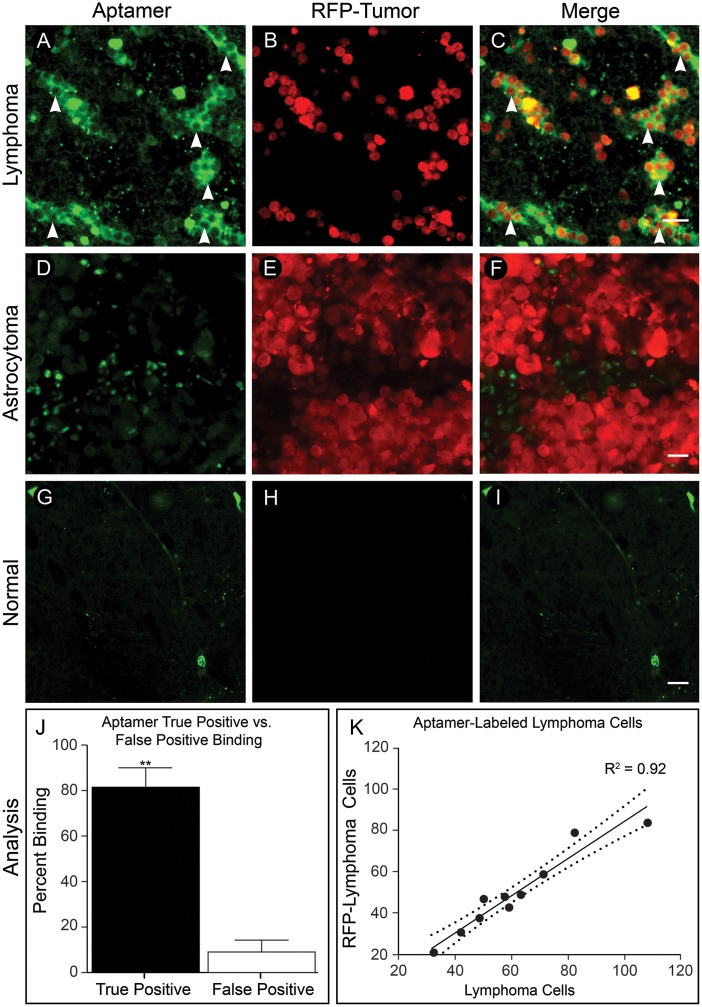
Fluorescent xenograft acute slices. (A-C) Lymphoma. A) Aptamer staining of lymphoma acute slice with positive staining regions (arrowheads). B) RFP-expressing lymphoma cells within tissue slice. C) Merge (D-F) Astrocytoma. D) Aptamer staining of astrocytoma acute slice; note lack of positively stained cells. E) RFP-expressing astrocytoma cells within tissue slice. F) Merge. (G-I) Normal brain. G) Aptamer staining of normal brain lacking aptamer-positive cells. H) Absence of ring-like staining and RFP-expressing cells in normal brain. I) Merge. J) Q-TD05 labels 80.75 ± 2.52% of RFP-expressing lymphoma cells and 8.25 ± 1.51% of non-RFP cells, p<0.001. K) Coefficient of correlation between RFP-lymphoma cells and aptamer staining (R^2^) = 0.92 with 95% confidence intervals (dotted lines) of 0.87–0.99, p<0.001. Scale bars equal 20*um*. © 2015, Barrow Neurological Institute, provided under CC BY 4.0.

To visualize the localization of Q-TD05 and examine the labeling accuracy, we produced rodent xenografts with human B cell lymphoma and astrocytoma cells expressing red fluorescent protein (RFP) (n = 4 lymphoma and 3 astrocytoma slices). Fresh acute slices generated from these animals were incubated with Q-TD05 for one hour and imaged without rinsing. We found strong circumferential staining of RFP-labeled lymphoma cells with Q-TD05 (Fig 4A–4C). Acute slices from astrocytoma xenografts showed strong RFP expression but lacked circumferential staining (Fig 4D–4F). As an additional negative control, we did not find fluorescently labeled cells in acute slice normal brain regions (Fig 4G–4I). We quantified labeling of Q-TD05 to RFP-labeled lymphoma cells (n = 13 fields of view from 3 acute slices). We found Q-TD05 labeled a significantly greater percentage of RFP-labeled lymphoma cells than non-RFP labeled cells (80.75 ± 2.52% vs. 8.25 ± 1.51%, p<0.001), and a strong correlation between aptamer staining and RFP-expressing lymphoma cells (R^2^ = 0.92, p<0.001) (Fig 4J and 4K).

## Discussion

We have demonstrated the potential of a FRET-based aptamer for providing antibody-specific diagnostic information in a small fraction of the time required for traditional IHC in cell culture and in an animal model. Specifically, we tested the specificity of Q-TD05 in human cell culture and tissues from orthotopic rodent xenografts. Compared with the current standard of CD20 IHC, Q-TD05 provided significantly faster identification of lymphoma cells with a less complex staining procedure (Supplemental Fig 1). Coupled with fluorescence imaging, Q-TD05 allowed rapid differentiation of human lymphoma cells from a clinically meaningful negative control of human astrocytoma based on staining intensity and morphology.

Antibody staining can be a rate-limiting step for definitive surgical treatment of cancers. When intraoperative histopathological diagnostics cannot differentiate cancers with opposing treatment paradigms such as CNS lymphoma versus astrocytoma, antibody staining is utilized to differentiate tissue based on tumor-specific protein expression [3]. This method requires numerous steps for tissue preparation and staining, and therefore typically requires at least 24–48 hours for processing [2, 6]. In contrast, our Q-TD05 aptamer identified CD20-positive tumor cells in fresh and fixed tissue with a one-step staining protocol with no special tissue preparation. This was evident in tumor core regions and tumor margins which contain more heterogenous cell populations (S1 Fig) [21]. Q-TD05 allowed antibody-like specific detection of CNS lymphoma cells within 15–60 minutes. Though labeling with this proof-of-concept quenchable aptamer exceeds the frozen section time range, it specifically labels lymphoma cells nearly two orders of magnitude faster than IHC. Additionally, development of aptamers with enhanced binding affinities is currently underway and could further decrease this staining time.

Advancements in optical imaging and development of fluorescent probes have provided researchers powerful tools for characterizing tissue, and we believe great advances will be made by applying these tools to intraoperative tissue diagnostics. The ability to make rapid and specific intraoperative diagnoses could decrease surgical times, provide additional guidance to surgeons in the operating suite, decrease the time patients are required to wait for a definitive diagnosis, and potentially decrease healthcare costs by facilitating and expediting diagnosis-based treatment decisions. Though the initial cost of fluorescence imaging equipment may be an obstacle to widespread use of switchable-aptamers, the one-step staining protocol and rapid diagnostic output could justify the expense, making them cost-effective with even moderate use. The attractiveness of much more rapid definitive pathological results will likely drive adoption. In addition, current efforts to miniaturize and economize fluorescence imaging instruments will soon make this imaging modality a realistic option for many pathology departments [22, 23].

## Conclusion

We have demonstrated that a conformational FRET-based switchable aptamer targeting human B cell lymphoma can rapidly and specifically identify tumor cells in biopsies from rodent xenograft models of human brain tumors. Translated into clinical pathology, quenchable aptamers have potential for providing intraoperative diagnoses not currently possible with frozen sections. Development of these aptamers against additional clinical targets could provide specific diagnoses in timeframes that support intraoperative decision-making and facilitate clinical management of patients whose treatment depends on their pathological diagnosis.

## Supporting Information

S1 Fig Colabeling: TD05 and CD20 antibody. (A and D) Aptamer. B cell lymphoma xenograft slices incubated with the quenchable aptamer; 1 hour staining. (B and E) CD20 antibody. B cell lymphoma slices incubated with CD20 antibody; 24 hour staining time. (C and F) Merge. Merged image of aptamer and CD20 antibody staining. (G) Spectral analysis. Ring-like staining patterns contain strong 488*nm* and 594*nm* fluorescence emissions; wavelengths unique to Q-TD05 and CD20 antibody. Scale bars equal 20*μm*. © 2015, Barrow Neurological Institute, provided under CC BY 4.0.
(TIF)

S1 Table Quantification of probe staining profile in cultured human B lymphoma cells (Ramos, the CD20 positive cell line) and T lymphoma cells (Jurkat, the CD20 negative control cell line). The percentage of the positively stained population in each cell line is listed in the table, and the signal/noise ratios are shown as the ratio between the percentage of positively stained population in Ramos and Jurkat cells.
(DOCX)
