# Supplementary figures and images for: Use of a Conformational Switching Aptamer for Rapid and Specific Ex Vivo Identification of Central Nervous System Lymphoma in a Xenograft Model
Source: PLoS One. 2015 Apr 15;10(4):e0123607. doi: 10.1371/journal.pone.0123607 (PMC4398547; doi:10.1371/journal.pone.0123607)

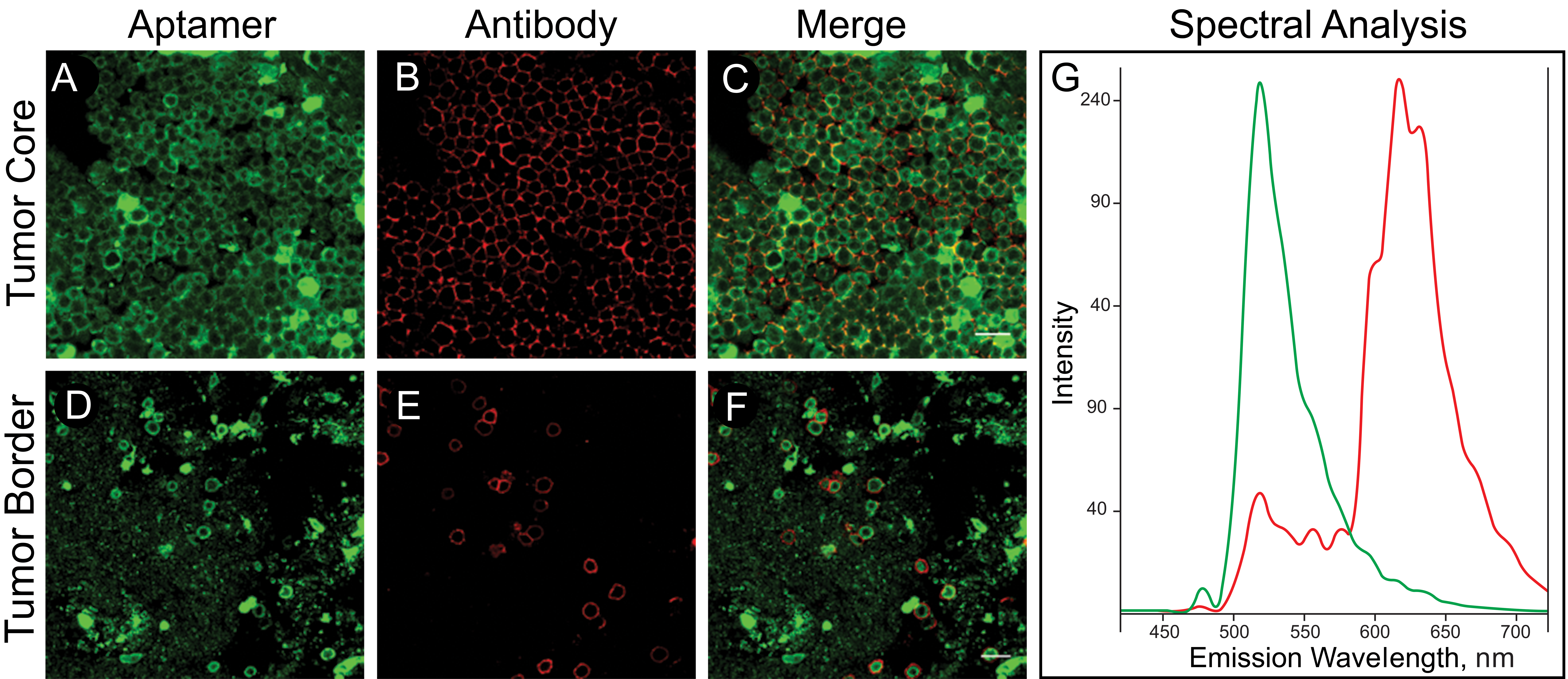

Supplement: S1 Fig — (A and D) Aptamer. B cell lymphoma xenograft slices incubated with the quenchable aptamer; 1 hour staining. (B and E) CD20 antibody. B cell lymphoma slices incubated with CD20 antibody; 24 hour staining time. (C and F) Merge. Merged image of aptamer and CD20 antibody staining. (G) Spectral analysis. Ring-like staining patterns contain strong 488nm and 594nm fluorescence emissions; wavelengths unique to Q-TD05 and CD20 antibody. Scale bars equal 20μm. © 2015, Barrow Neurological Institute, provided under CC BY 4.0. (TIF) [file pone.0123607.s001.tif]
