# Supplementary material for: Use of a Conformational Switching Aptamer for Rapid and Specific Ex Vivo Identification of Central Nervous System Lymphoma in a Xenograft Model
Source: PLoS One. 2015 Apr 15;10(4):e0123607. doi: 10.1371/journal.pone.0123607 (PMC4398547; doi:10.1371/journal.pone.0123607)
Supplement: S1 Table — The percentage of the positively stained population in each cell line is listed in the table, and the signal/noise ratios are shown as the ratio between the percentage of positively stained population in Ramos and Jurkat cells. (DOCX) [file pone.0123607.s002.docx]

**S1 Table.** Quantification of probe staining profile in cultured human B lymphoma cells (Ramos, the CD20 positive cell line) and T lymphoma cells (Jurkat, the CD20 negative control cell line). The percentage of the positively stained population in each cell line is listed in the table, and the signal/noise ratios are shown as the ratio between the percentage of positively stained population in Ramos and Jurkat cells.

| **Probe** | **Positive in Ramos (%)** | **Positive in Jurkat (%)** | **Signal/noise**  **(Raoms/Jurkat)** |
| --- | --- | --- | --- |
| Switchable | 94.14 | 11.77 | 8.00 |
| Always On | 99.91 | 81.90 | 1.22 |
